# Treatment of Enlarged Bursæ by Caustics

**Published:** 1857-11

**Authors:** Jas. B. Prowse

**Affiliations:** Clifton


*Treatment of Enlarged Bursæ by Caustics. By Jas. B. Prowse, Esq., M.R.C.S., Clifton.*

I have been in the habit of removing enlarged bursæ by means of the lunar caustic, applied in the form of the stick moistened with a little water and rubbed over the whole surface of the enlargement for the space of some minutes. In most cases this treatment will be found effectual where the blisters, the tincture of iodine, etc., have been but of little use, and it will often obviate the necessity of passing setons through the tumor, or of removing it by means of dissection. Having in a great number of cases proved the efficacy of this plan, I can confidently recommend it for adoption in practice.—*London Lancet.*

